# 4q25 Microdeletion with Axenfeld-Rieger Syndrome and Developmental Delay

**DOI:** 10.1155/2023/4592114

**Published:** 2023-02-09

**Authors:** Yukino Kawanami, Tomoko Horinouchi, Naoya Morisada, Takeshi Kato, Kandai Nozu

**Affiliations:** ^1^Department of Pediatrics, Kobe University Graduate School of Medicine, Kobe, Japan; ^2^Department of Clinical Genetics, Hyogo Prefectural Kobe Children's Hospital, Kobe, Japan; ^3^Western Pediatric and Rehabilitation Center for the Disabled, Kobe, Japan

## Abstract

We encountered a case with congenital iris coloboma, omphalocele, and developmental delay with a 2.5 Mb deletion on chromosome 4q25 encompassing *PITX2*, leading to Axenfeld-Rieger syndrome (ARS)*, NEUROG2*, and *ANK2*. ARS is characterized by the aplasia of the anterior eye, odontogenesis, and abdominal wall aplasia. In our case, iris coloboma and omphalocele were thought to be caused by *PITX2* haploinsufficiency. However, these symptoms are nonspecific, and clinical symptoms alone can make it difficult to make a correct diagnosis. In addition, the genes responsible for developmental delay, among others, are not well understood. Developmental delay, in this case, might be caused due to *NEUROG2* haploinsufficiency. In spite of the partial deletion of *ANK2*, the causative gene of long QT syndrome type 4, the electrocardiogram was normal. Genetic testing can lead to a correct diagnosis, and it may be effective in detecting complications.

## 1. Introduction

Haploinsufficiency of multiple genes due to chromosomal microdeletion is known to cause various symptoms such as DiGeorge syndrome (MIM #188400), Williams-Beuren syndrome (MIM #194050), and Smith-Magenis syndrome (MIM #182290) with strong clinical manifestations [1]. Recent advances in microarray analysis have led to the identification of many other causative genes and regions. With the exception of a few reports on 4q25 microdeletion that did not include the *PITX2* gene region [2, 3], 4q25 microdeletion is mainly a case of Axenfeld-Rieger syndrome (ARS) caused by abnormalities in the *PITX2* gene (MIM# 601542) [4]. *PITX2* encodes paired-like homeodomain transcription factor 2, which is involved in the development of eyes, teeth, and abdominal organs [5]. Although ARS has multiple systemic symptoms and some case reports have been made [6–8], its diagnosis based on clinical symptoms alone is not easy. In this study, we reported a case of multiple malformations and developmental delay diagnosed with a 4q25 microdeletion leading to ARS.

## 2. Clinical Report

**Figure 1:**
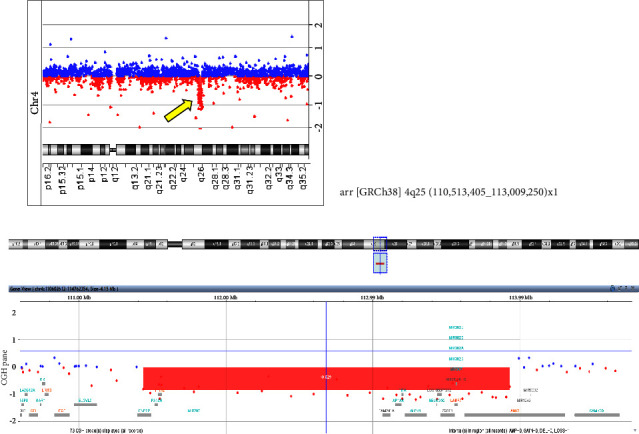
Array-based comparative genomic hybridization analysis revealed 2.5 Mb deletion on chromosome 4q25, arr[GRCh38] 4q25(110,513,405_113,009,250)x1 encompassing *ENPEP*, *PANCR*, *PITX2, MIR297*, *FAM241A*, *AP1AR*, *TIFA, ALPK1, NEUROG2, LOC105377372*, *ZGRF1*, *LARP7*, *MIR302CHG*, *MIR367*, *MIR302D*, *MIR302A*, *MIR302C*, *MIR302B*, and *ANK2.*

A 3-year-old boy was born to healthy nonconsanguineous parents at 37 weeks of gestation. He was born with a birth weight of 2,494 g (33.2 percentile), a head circumference of 34 cm (84.0 percentile), a length of 44.5 cm (10.9 percentile), and a chest circumference of 28 cm. The Apgar score was 8 at 1 min. The patient was the only child of his parents, and he had no family history. He was born with a congenital omphalocele, which was immediately repaired after birth. Moreover, congenital iris coloboma, astigmatism, and exotropia were diagnosed, and the patient underwent regular check-ups with a pediatric ophthalmologist. At one year of age, he started rehabilitation due to delayed development; he could not stand or speak significant words. Additionally, shortening of the lingual frenulum and upper labial frenulum was observed. The patient was referred to our hospital for genetic testing. An array-based comparative genomic hybridization analysis was performed after obtaining written informed consent from the parents. We identified a 2.5-Mb deletion on chromosome 4q25, arr[GRCh38] 4q25(110,513,405_113,009,250)x1 (Figure 1). The deletions encompassed *ENPEP*, *PANCR*, *PITX2*, *MIR297*, *FAM241A, AP1AR*, *TIFA, ALPK1*, *NEUROG2, LOC105377372*, *ZGRF1*, *LARP7*, *MIR302CHG*, *MIR367, MIR302D*, *MIR302A*, *MIR302C, MIR302B,* and *ANK2.* As *ANK2* is known to cause long QT syndrome (LQTS) type 4 (MIM# 600919), we consulted a pediatric cardiologist and performed electrocardiography; however, no abnormalities were noted this time.

## 3. Discussion

ARS (MIM# 180500) is a clinically and genetically heterogeneous autosomal dominant rare disease with 40–70% abnormalities in *PITX2* (4q25) or *FOXC1* (6p25); however, abnormalities in other genes such as *PAX6*, *CYP1B1*, and *PRDM5* have also been reported [9, 10]. Abnormal ectoderm formation associated with an abnormal transcription factor is the leading cause of the disorder, and its symptoms include aplasia of the anterior eye, odontogenesis, and abdominal wall aplasia [11, 12]. ARS associated with *PITX2* abnormalities has been reported to have more extraocular complications than ARS with *FOXC1* abnormalities [13, 14]; however, its symptoms are nonspecific, and its clinical manifestations are not easy to diagnose. Additionally, in ARS cases, the risk of glaucoma is particularly high, and patients need to follow up closely [12]. Therefore, an accurate diagnosis using genetic testing is very important.

**Figure 2:**
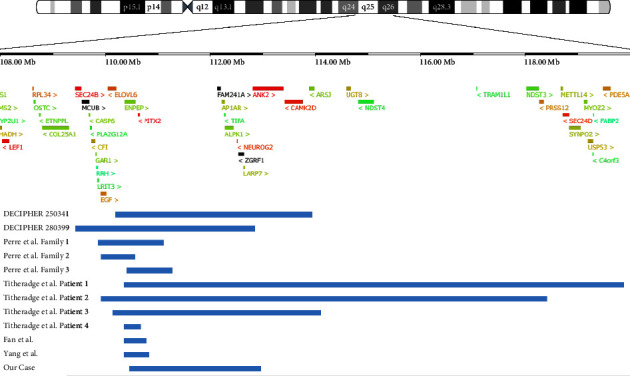
Reported cases with microdeletions of less than 10 Mb encompassing *PITX2* are shown. Blue lines show the range of deletions. Protein-coding genes colored by pHaplo scores were downloaded from the DECIPHER browser [17] (https://www.deciphergenomics.org/search/patients/browser?q=4%3A108000000-120000000, accessed on 25 December 2022).

Some studies have been conducted to determine the spectrum of symptoms associated with 4q25 microdeletion; however, further clarification is required [4, 15, 16]. The main symptom in patients with 4q25 microdeletion is ARS associated with *PITX2*. Reported cases in which detailed positions are shown for microdeletions of less than 10-Mb, encompassing *PITX2*, with phenotypes older than 1 year, were summarized in Figure 2 and Table 1 [17]. The probability of being loss-of-function intolerant (pLI) score partitions genes into loss-of-function intolerant (pLI ≥ 0.9) or tolerant (pLI ≤ 0.1) [18]. Table 1 lists the genes with pLI scores of 0.6 or more. As for eye lesions, tooth defects, and umbilical problems, *PITX2* seems to be the cause, as with ARS. However, some studies have reported cases of developmental and psychomotor delay, suggesting the involvement of contiguous genes [4, 19]. Among the contiguous genes, *NEUROG2*, *UGT8*, *NDST3*, and *PRSS1* are candidate genes associated with neurodevelopmental phenotypes [4]. Our cases, DECIPHER 250341 and DECIPHER 280399, encompass a deletion in *NEUROG2*, which may be related to intellectual problems and/or psychomotor delays. Previously, Sterhle et al. have indicated the possible association between *NEUROG2* and neurologic findings [16]. Furthermore, the pLI score was 0.81 (https://gnomad.broadinstitute.org/gene/ENSG00000178403?dataset=gnomad_r2_1, accessed on 20 February 2022). The significance of the haploinsufficiency in *NEUROG2* needs further examination. Recent advances in microarray technology have revealed more detailed deletion sites, which may reveal associations between causative genes and symptoms.

*ANK2* is the causative gene for autosomal dominant LQTS type 4 [20]. Although some studies have reported LQTS cases involving translocation, including 4q25 microdeletion [21], no study has reported LQTS cases associated with ARS induced by 4q25 microdeletion. In the present case, electrocardiographic screening revealed no abnormalities. However, variants in *ANK2* can lead to not only LQTS but also sudden cardiac death, sinus node disease, arterial fibrillation, ventricular tachycardia, bradycardia, syncope, and arrhythmogenic right ventricular cardiomyopathy, all of which are critical and life-threatening [22]. It is beneficial to explain the risk of cardiac events to family members and encourage regular follow-up.

In conclusion, we encountered a Japanese case of 4q25 microdeletion with ARS, developmental delay, and the absence of LQTS. Even in cases where clinical diagnosis is difficult, genetic testing can help correct the diagnosis, and it may be effective in detecting complications and guiding appropriate follow-up. Further studies are needed to determine the phenotypes of the microdeletions.

## Figures and Tables

**Table 1:** Deletion site and clinical futures of the reported cases.

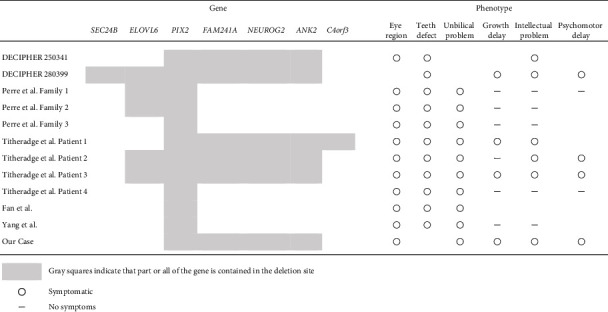

## Data Availability

The data supporting the findings of this study can be obtained from the corresponding authors on reasonable request.
